# Potential clinical application of anti-Müllerian hormone testing in radioiodine treatment of thyroid carcinoma

**DOI:** 10.1186/s13104-024-06802-y

**Published:** 2024-05-23

**Authors:** Barbora Havlínová, Ilona Součková, Kateřina Kopřivová, Jiří Doležal

**Affiliations:** 1 The 4th Department of Internal Medicine – Hematology, Faculty of Medicine in Hradec Králové, University Hospital Hradec Králové and Charles University, Sokolská 581, Hradec Králové, 50005 Czech Republic; 2 Department of Clinical Immunology and Allergology, Faculty of Medicine in Hradec Králové, University Hospital Hradec Králové and Charles University, Hradec Králové, Czech Republic; 3 Department of Nuclear Medicine, Faculty of Medicine in Hradec Králové, University Hospital Hradec Králové and Charles University, Hradec Králové, Czech Republic

**Keywords:** Ovarian reserve, Anti-Müllerian hormone, Radioiodine treatment of thyroid cancer, Premature ovarian failure

## Abstract

Differentiated thyroid cancer is the most common endocrinological malignancy. Radioiodine treatment has a clear benefit in locally aggressive and metastatic cancers. There are discussions about long-term and acute adverse events.

Anti-Müllerian hormone is regarded as the best endocrine marker for evaluating the physiological loss of oocytes in healthy women with regard to age. The impact of radioiodine treatment on anti-Müllerian hormone levels has been more significantly reported in patients over 35 years of age. About reproductive dysfunction, calculations of individual absorbed doses of radioiodine in ovaries after thyroid cancer therapy have not been performed yet. The aim of our ongoing prospective study is to determine serum anti-Müllerian hormone to estimate ovarian reserve for premenopausal women treated with radioiodine and to compare anti-Müllerian hormone levels before and after radioiodine treatment. Predicting radioiodine side effects by evaluating a simple serum biomarker may help to select an appropriate treatment strategy for young women planning pregnancy, specifically in the assessment of ovarian reserve and premature ovarian failure with early onset of menopause.

## Background

Anti-Müllerian hormone (AMH, anti-Müllerian hormone; MIH, Müllerian inhibitory hormone) is starting to be used as one of the main biomarkers of ovarian reserve. The ovarian reserve is the population of primordial (non-growing) follicles in the ovary [1]. The reserve size can be used to estimate reproductive functions for premenopausal women. Premature ovarian failure occurs as a result of depletion of the follicular reserve. Loss of ovarian function and early onset of menopause before age 40 affects 1% f women worldwide and may be associated with autoimmune disease (e.g. Graves-Basedow thyrotoxicosis, Hashimoto’s thyroiditis, type 1 diabetes mellitus) and/or genetic predisposition, including known incidence in congenital disorders such as Turner syndrome and X chromosome fragmentation syndrome. Low levels of anti-Müllerian hormone (AMH < 0.5 ng/mL), estradiol (E2 < 20 pg/mL), as well as a decrease in inhibin B and an increase in gonadotropins (follicle-stimulating hormone, FSH > 40 IU/L) are conclusive markers for the diagnosis of ovarian depletion [2]. AMH is considered an excellent marker of ovarian reserve due to the stability of its serum levels and the possibility of determination without affecting the result at any stage of the menstrual cycle [3].

**AMH** is a glycoprotein dimer with a molecular weight of 140 kDa belonging to the group of growth and differentiation factors (TGF-β, transforming growth factor-β). It is encoded on chromosome 19 (19p13.2–13.3). The signal pathway is transmembrane mediated by the dimeric serine/threonine kinase receptor. AMH influences prenatal fetal development and sex differentiation along with other hormonal factors, signaling molecules, and genetic factors (e.g. SF1 gene, DAX1, SRY) [4].

In the presence of AMH, the Müllerian ducts regress, thereby inhibiting the development of the uterus, fallopian tubes, and upper part of the vagina. It also has a regulatory role in the development and maturation of the gonads after birth. In males, AMH is formed in the Sertoli cells of the testes most prenatally, from 8 to 9 weeks after conception, decreases markedly with the onset of puberty as testosterone production increases, and in adulthood the level of AMH remains physiologically low. Compared *to* female babies, AMH begins forming around 36 weeks of gestation (around the time of birth) in ovarian granulosa cells of growing follicles, peaks at around the age of 25, and continues to decline until menopause [5, 6].

From the total number of primary oocytes produced (1–2 × 10^6^), a maximum of 400–500 germ cells are released during the reproductive period, with the remaining germ cells undergoing atresia [7]. In the postnatal ovary, AMH is produced in the granulosa cells of growing follicles from the primary to the early antral stage, it is secreted by primary, secondary, pre-antral, and small antral follicles sized < 4 mm [6, 8].

Treatment with radioiodine (RAI, I-131) has more than 60 years of tradition. Its therapeutic effect in patients with differentiated thyroid cancer at high risk of recurrence has been unequivocally demonstrated during this time. In patients with low-risk cancer, the positive effect of radioiodine administration on induction of remission and overall survival has not been unequivocally demonstrated in recent prospective clinical trials. Systemic adjuvant therapy I-131 (mixed β and γ emitter) is administered for the radio-elimination of post-operative thyroid remnants as well as unresectable or incompletely resected thyroid tissue.

With RAI, we are able to detect local recurrence or distant metastases in the early phase of the disease, which contributes to treatment success and minimization of mortality. Regional or distant metastases are often also detectable with rising thyroglobulin levels after successful thyroid ablation of thyroid remnants. A brief overview of radioiodine treatment and other therapeutic procedures are summarized in Table 1. In the period of pregnancy and lactation, the administration of I-131 is contraindicated [6, 9]. The absorbed dose of RAI in organs is not routinely calculated.

**Table 1:** Concise overview of treatment procedure for thyroid cancer

Therapeutic procedure	Comment
1. Surgical removal of the thyroid gland – hemithyroidectomy or total thyroidectomy according to the risk (histological variant and grade of the carcinoma, clinical stage)	Differentiated carcinomas: follicular, papillary, oncocytic (recognised as a separate entity or as a variant of follicular carcinoma); undifferentiated carcinomas: medullary, anaplastic; microcarcinoma (tumour up to 20 mm in the largest diameter)
2. Thyroid-stimulating hormone (TSH) suppression therapy or subsequent radioiodine therapy to destroy postoperative residues – only relevant for tumours with the ability to uptake/accumulate radioiodine	Repeated administration of RAI – in case of early detected local cancer recurrence – without/with metastatic lymph node involvement, if there is no indication for surgical removal; multiple administrations are possible, usually at a higher RAI dose (cumulative dose calculation) the ability to absorb RAI may be lost after repeated applications and during cancer dedifferentiation
3. Follow-up at regular intervals in the Nuclear Medicine Department – regular laboratory checks, neck ultrasound, ^99^Tc-MIBI scintigraphy	Laboratory marker of recurrence: thyroglobulin, therapeutic target – suppression of TSH

This local clinical study, conducted at the University Hospital in Hradec Králové, determines serum AMH levels and estimates ovarian reserve for premenopausal women treated with radioiodine. The objective is to mitigate adverse effects of radioisotope therapy for young women with risk factors (e.g. gynaecological history of PCOS, endometriosis, fibroids, gynaecological tumours, breast cancer, associated internal diseases) and possibly to provide individual consideration on the need for RAI administration.

Aims of the project are:

1. to determine the absorbed dose of ionizing radiation in the ovaries of women undergoing radioiodine treatment based on the magnitude of I-131 activity administered,
2. to determine ovarian reserve assessed by AMH,
3. to evaluate the relationship between absorbed dose of I-131 and change in AMH level.

## Materials and methods

### Methods of determination of AMH and its serum concentration

AMH is involved in the development of gonads and regulates their maturation after birth. The main physiological role of AMH is aimed at inhibiting the recruitment of primary follicles, thereby preventing follicular reserve depletion too early – it acts as an antagonist to FSH. In vivo and in vitro experiments have shown that the transition from primary to growing follicles is enhanced without AMH [10].

The determination of ovarian reserve by AMH examination is mainly used for in vitro fertilization methods, as well as for prediction of the onset of menopause and thus estimation of the length of the reproductive period [4]. Additionally, it is employed in the diagnosis and monitoring of polycystic ovarian syndrome (PCOS), as well as in diagnosing and treating ovarian granulosa cell tumors. Other indications include diagnosis and differential diagnosis of premature and delayed puberty in boys, diagnosis of cryptorchism and anorchism in boys, and differential diagnosis of impaired sexual development in children [11].

Methods for the determination of AMH are not standardized owing to little-studied factors affecting its serum levels (intraindividual differences, age, body constitution, genetic factors, associated diseases and pharmacotherapy) [12]. It is recognized that as women age, AMH levels progressively decline to undetectable levels, reflecting the diminishing number of follicles in the ovaries. Another variability is polycystic ovary syndrome, where higher AMH concentrations occur at an older age. AMH as the only determining fertility marker is not used independently yet due to the lack of data from conducted studies so far.

In current clinical practice, a minimal assessment of AMH, follicle-stimulating hormone, estradiol, and inhibin B is established. There are different interpretations of serum AMH levels – standards and threshold values vary. AMH is found in the serum in the inactive uncleaved form of pro-AMH and the cleaved, biologically active form of AMH (AMHN, AMHC). The biologically active AMH molecule is composed of two N-terminal and two C-terminal non-covalently associated fragments that bind to AMH receptors type II (AMH RII). Pro-AMH and AMH (AMHN, AMHC) are detected by immunological methods [13]. In sandwich ELISA (Enzyme-Linked ImmunoSorbent Assay) detection, 2 antibodies, capture, and detection, bound to the N-terminal and C-terminal parts of AMH. A physiological range of serum AMH values of 2–6.8 ng/mL is indicated and indicates a sufficient number of eggs and therefore optimal fertility. It also appears to be a good marker due to its stability of serum levels and the possibility of determining them without affecting the result in any phase of the menstrual cycle [3]. As a woman ages, it gradually decreases to unmeasurable values. Changes in serum AMH levels depending on the amount of administered I-131 activities are poorly described, and there are no available studies evaluating AMH changes in connection with absorbed doses of radioiodine in the ovaries.

The proposed study protocol specifies to collect biological material (venous blood) from a selected group of patients before administration of RAI and again after radioiodine administration at regular intervals. The target group of the study is female patients of childbearing age (mean 35.5 years) treated for thyroid cancer. Collection of biological material (venous blood) is performed after obtaining informed consent in a selected group of patients. The objectives are gonadotropic axis examination, thyroid function testing and assessment of AMH changes before and after RAI administration at regular intervals as per protocol schedule at 3–4 months, 6–8 months, 12 months and 18 months after RAI administration.

Each patient provides a total of 5 samples. The correlation between AMH and sex hormones, including thyroid function, is also evaluated. In each sample, the following are measured: AMH, gonadotropins and dehydroepiandrosterone, serum proteins with sex hormone binding globulins, thyroid hormones and thyroglobulin with antibodies, baseline mineralogram, liver enzymes, screening for type 2 diabetes mellitus, and renal function. The control group consists of 3 healthy female patients (aged 28–35 years) in whom AMH and gonadotropic axis function are compared at an interval of one year.

Collected AMH samples are stored by freezing for subsequent measure in serum and plasma by ELISA using the AMH Gen II ELISA kit (one kit designed to test 80 samples by sandwich detection). Reference ranges for AMH levels are set by the ELISA kit manufacturer (Beckman Coulter). The normalized absorbed dose in the ovaries for the reference patient, according to the International Commission on Radiological Protection (ICRP) and ICRP Publication 128 (2014), is 0.036 mGy/MBq. The absorbed radioiodine dose can be calculated from the administered ionizing radiation activity using software like Dosewatch (GE Healthcare). This is a study design, all results are not available yet.

## Discussion

Differentiated thyroid carcinoma (DTC) is the most common endocrinological malignancy, the incidence of which has been increasing in the last 30 years. It is the fifth most common tumor in the young population (15–39 years). DTC is usually diagnosed in women aged 30–39 years. Differentiated papillary and follicular carcinomas account for the largest proportion of all thyroid cancers. In indicated cases, the treatment method is surgical removal of the tumor followed by adjuvant administration of RAI. An excellent survival prognosis is documented for differentiated carcinomas. Five-year survival approaches 100% for localized disease and 96% is reported for loco-regional disease with metastatic lymph node involvement. Total thyroidectomy followed by RAI is recommended for all patients with DTC > 1 cm, despite international trends for less aggressive treatment. Currently, there is a debate about what should be the smallest amount of radioiodine activity administered to achieve complete elimination of postoperative residues. The German Society of Nuclear Medicine DGN (Die Deutsche Gesellschaft für Nuklearmedizin) recommends an optimal ablation activity value of 1–3.7 GBq (about 30–100 mCi) of I-131 [9].

Treatment with radioiodine is generally well tolerated by patients. However, some short-term and long-term side effects may occur [14]. Temporary side effects include radiation thyroiditis, local swelling of the accumulating RAI in foci or metastases, gastritis and nausea, sialoadenitis, and taste and smell abnormalities. Depending on the magnitude of radioiodine activity and cumulative doses administered, bone marrow attenuation, hypospermia, and menstrual cycle disturbances may occur. In the long term, fertility is reported to be unchanged in both men and women. Long-term side effects include the development of subsequent secondary malignancies (leukemia and solid tumors), chronic sialoadenitis, taste and smell abnormalities, xerostomia and pulmonary fibrosis [6]. Despite many years of therapy and data from studies conducted, radioiodine is considered a safe treatment modality.

There are currently many studies investigating AMH to find an ovarian reserve for in vitro fertilization methods. AMH testing is gaining in importance in current clinical practice. International works have cited AMH as the best endocrine marker for assessing age-related oocyte physiological loss for healthy women. AMH as a fertility marker is not used alone. Minimal assessment of AMH, follicle-stimulating hormone, estradiol and inhibin B is established. Variations in serum AMH levels may be due to the action of various factors including combinations of factors (e.g., intra-individual differences, age, body constitution, genetic factors, race/ethnicity, associated diseases, drug therapy) that have not been adequately investigated [13]. Despite this, AMH appears to be a unique endocrine parameter in the investigation of ovarian reserve. Indeed, levels of AMH are stable at any stage of the menstrual cycle, are not affected by the use of oral contraceptives, and even do not change during pregnancy, when the release of endogenous gonadotropins is substantially reduced. These findings are consistent with the idea that AMH levels reflect continuous FSH independent non-cyclical growth of small follicles in the ovary [3].

The effect of RAI treatment on AMH levels has been more significantly observed in patients over 35 years of age. Although meta-analyses of data from retrospective studies showed a significant decrease in AMH levels after treatment of DTC with radioiodine, there was no evidence of a long-term decrease in pregnancy rates [15]. However, further prospective studies with more data should be conducted to verify these results. The physiological decline of reproductive functions over time with increasing age of the woman should be taken into consideration.

In connection with reproductive disorders, calculations of absorbed doses of radioiodine to the ovaries of patients after thyroid cancer therapy have not been performed yet.

In future studies, it is important to focus on the exact design of the study and the division of patients undergoing RAI treatment into predefined groups (age, risk factors). The aim should be to avoid adverse effects of radioisotope treatment for young women with risk factors (gynecological history of PCOS, endometriosis, myomas, gynecological tumors, breast cancer and associated internal diseases) and possibly individual consideration of the need for RAI administration. Thus, the rationale of the indication, the exact description of the treatment, the nature of the disease, prognosis, and treatment response.

## Limitations

This study exhibits the following limitations:

This is a design of prospective study and the complete results are not available yet.

Laboratory blood tests (study protocol) were performed in a small sample of patients (*n* = 33) with thyroid cancer indicated for radioiodine treatment. Changes in AMH levels should be further compared at repeated intervals after radioiodine treatment.

The study spans across a long time due to the need for repeated hormone profile testing before and after treatment with I-131 at regular intervals, consideration of the physiological loss of oocytes in healthy women with regard to age, examination of a control group of healthy patients.

Subsequent research aims to evaluate the qualitative insights leaned from this study in larger and more representative samples (young patients in reproductive age).

## Conclusion

The safety of the administered dose of radioiodine can be verified by calculating the absorbed ionizing radiation by the reproductive organs and by examining a simple serum biomarker.

Predicting adverse effects may help to select an appropriate treatment strategy for young women planning pregnancy.

## Data Availability

No datasets were generated or analysed during the current study.

## Abbreviations

AMH: Anti-Müllerian hormone
MIH: Müllerian inhibitory hormone
E2: Estradiol
FSH: Follicle-stimulating hormone
TGF-β: Transforming growth factor-β
SF-1: Steroidogenic factor 1
DAX1: Dosage-sensitive sex reversal, adrenal hypoplasia critical region, on chromosome X, gene 1
SRY: Sex-determining region Y
RAI: Radioiodine, I-131
PCOS: Polycystic ovary syndrome
AMHN: N-terminal homodimer of anti-Müllerian hormone
AMHC: C-terminal homodimer of anti-Müllerian hormone
AMHR2: Anti-Müllerian hormone receptor type 2
ELISA: Enzyme-Linked ImmunoSorbent Assay
DTC: Differentiated thyroid carcinoma
TSH: Thyroid-stimulating hormone
